# Supplementary material for: Modeling glioblastoma heterogeneity as a dynamic network of cell states
Source: Mol Syst Biol. 2021 Sep 16;17(9):e10105. doi: 10.15252/msb.202010105 (PMC8444284; doi:10.15252/msb.202010105)
Supplement: Supplementary file 6 — Source Data for Figure 5 [file MSB-17-e10105-s004.zip › Figure5A_sourcedata/GSEA_3071/nosigdb_stateIV.GseaPreranked.1622795164626/VANGRONINGEN_ADRN.html]

Details for gene set VANGRONINGEN\_ADRN[GSEA]

|  || Dataset | state53071 |
| Phenotype | NoPhenotypeAvailable |
| Upregulated in class | na\_pos |
| GeneSet | VANGRONINGEN\_ADRN |
| Enrichment Score (ES) | 0.45098937 |
| Normalized Enrichment Score (NES) | 2.7873755 |
| Nominal p-value | 0.0 |
| FDR q-value | 0.0 |
| FWER p-Value | 0.0 |
Table: GSEA Results Summary

  

Fig 1: Enrichment plot: VANGRONINGEN\_ADRN      
 Profile of the Running ES Score & Positions of GeneSet Members on the Rank Ordered List

  

| PROBE | GENE SYMBOL | GENE\_TITLE | RANK IN GENE LIST | RANK METRIC SCORE | RUNNING ES | CORE ENRICHMENT || 1 | DCX |  |  | 4 | 2.752 | 0.0174 | Yes |
| 2 | BEX1 |  |  | 18 | 2.252 | 0.0297 | Yes |
| 3 | NRCAM |  |  | 23 | 2.185 | 0.0434 | Yes |
| 4 | CADM1 |  |  | 38 | 1.965 | 0.0537 | Yes |
| 5 | INSM1 |  |  | 46 | 1.869 | 0.0646 | Yes |
| 6 | ASCL1 |  |  | 53 | 1.813 | 0.0754 | Yes |
| 7 | MAP2 |  |  | 55 | 1.811 | 0.0872 | Yes |
| 8 | SOX11 |  |  | 56 | 1.811 | 0.0992 | Yes |
| 9 | CELF2 |  |  | 63 | 1.766 | 0.1097 | Yes |
| 10 | TUBB2B |  |  | 69 | 1.731 | 0.1201 | Yes |
| 11 | TAGLN3 |  |  | 71 | 1.720 | 0.1313 | Yes |
| 12 | CKB |  |  | 72 | 1.711 | 0.1426 | Yes |
| 13 | DPYSL3 |  |  | 75 | 1.691 | 0.1534 | Yes |
| 14 | EML6 |  |  | 84 | 1.619 | 0.1625 | Yes |
| 15 | GAP43 |  |  | 120 | 1.487 | 0.1655 | Yes |
| 16 | NCAM1 |  |  | 129 | 1.456 | 0.1736 | Yes |
| 17 | TFAP2B |  |  | 134 | 1.433 | 0.1822 | Yes |
| 18 | CXADR |  |  | 139 | 1.402 | 0.1907 | Yes |
| 19 | TMTC4 |  |  | 147 | 1.382 | 0.1985 | Yes |
| 20 | GRIA2 |  |  | 160 | 1.347 | 0.2050 | Yes |
| 21 | TUBB3 |  |  | 167 | 1.330 | 0.2127 | Yes |
| 22 | IGSF3 |  |  | 173 | 1.309 | 0.2203 | Yes |
| 23 | NCAN |  |  | 181 | 1.298 | 0.2275 | Yes |
| 24 | CXCR4 |  |  | 197 | 1.269 | 0.2330 | Yes |
| 25 | PKIA |  |  | 204 | 1.253 | 0.2401 | Yes |
| 26 | SHC3 |  |  | 215 | 1.218 | 0.2462 | Yes |
| 27 | SATB1 |  |  | 222 | 1.203 | 0.2530 | Yes |
| 28 | MYEF2 |  |  | 225 | 1.196 | 0.2605 | Yes |
| 29 | FZD3 |  |  | 231 | 1.185 | 0.2673 | Yes |
| 30 | ATP6V0E2 |  |  | 236 | 1.179 | 0.2743 | Yes |
| 31 | KIF21A |  |  | 239 | 1.171 | 0.2817 | Yes |
| 32 | KIF5C |  |  | 240 | 1.170 | 0.2894 | Yes |
| 33 | FHOD3 |  |  | 245 | 1.161 | 0.2963 | Yes |
| 34 | TCEAL7 |  |  | 249 | 1.150 | 0.3033 | Yes |
| 35 | HES6 |  |  | 250 | 1.150 | 0.3109 | Yes |
| 36 | RBP1 |  |  | 254 | 1.136 | 0.3178 | Yes |
| 37 | DPYSL2 |  |  | 279 | 1.077 | 0.3203 | Yes |
| 38 | FOXO3 |  |  | 291 | 1.054 | 0.3251 | Yes |
| 39 | SCG2 |  |  | 294 | 1.040 | 0.3315 | Yes |
| 40 | CCSAP |  |  | 299 | 1.036 | 0.3376 | Yes |
| 41 | NMNAT2 |  |  | 302 | 1.034 | 0.3441 | Yes |
| 42 | DACH1 |  |  | 303 | 1.033 | 0.3509 | Yes |
| 43 | MSI2 |  |  | 354 | 0.971 | 0.3475 | Yes |
| 44 | RUFY3 |  |  | 376 | 0.929 | 0.3495 | Yes |
| 45 | PNMA2 |  |  | 390 | 0.915 | 0.3530 | Yes |
| 46 | ZNF91 |  |  | 392 | 0.913 | 0.3589 | Yes |
| 47 | ATCAY |  |  | 397 | 0.910 | 0.3641 | Yes |
| 48 | DPYSL5 |  |  | 411 | 0.895 | 0.3675 | Yes |
| 49 | KLHL13 |  |  | 412 | 0.893 | 0.3734 | Yes |
| 50 | RAB6B |  |  | 424 | 0.877 | 0.3770 | Yes |
| 51 | CRMP1 |  |  | 438 | 0.866 | 0.3802 | Yes |
| 52 | SBK1 |  |  | 439 | 0.865 | 0.3859 | Yes |
| 53 | EEF1A2 |  |  | 441 | 0.864 | 0.3914 | Yes |
| 54 | CYFIP2 |  |  | 450 | 0.856 | 0.3955 | Yes |
| 55 | ARL6IP1 |  |  | 458 | 0.849 | 0.3997 | Yes |
| 56 | RTN1 |  |  | 471 | 0.836 | 0.4029 | Yes |
| 57 | ALK |  |  | 477 | 0.832 | 0.4074 | Yes |
| 58 | ZNF704 |  |  | 498 | 0.818 | 0.4089 | Yes |
| 59 | ANK2 |  |  | 512 | 0.807 | 0.4117 | Yes |
| 60 | DUSP4 |  |  | 553 | 0.761 | 0.4089 | Yes |
| 61 | ELAVL3 |  |  | 582 | 0.739 | 0.4083 | Yes |
| 62 | TMOD2 |  |  | 591 | 0.732 | 0.4116 | Yes |
| 63 | RNF165 |  |  | 606 | 0.725 | 0.4136 | Yes |
| 64 | AP1S2 |  |  | 608 | 0.723 | 0.4182 | Yes |
| 65 | GLCCI1 |  |  | 611 | 0.719 | 0.4226 | Yes |
| 66 | GDAP1 |  |  | 617 | 0.715 | 0.4263 | Yes |
| 67 | LEPROTL1 |  |  | 624 | 0.712 | 0.4298 | Yes |
| 68 | SCG3 |  |  | 632 | 0.707 | 0.4331 | Yes |
| 69 | ATP6V1B2 |  |  | 674 | 0.674 | 0.4296 | Yes |
| 70 | BEX2 |  |  | 725 | 0.645 | 0.4240 | Yes |
| 71 | ANP32A |  |  | 731 | 0.643 | 0.4273 | Yes |
| 72 | C14orf132 |  |  | 758 | 0.624 | 0.4263 | Yes |
| 73 | PPP1R9A |  |  | 768 | 0.621 | 0.4287 | Yes |
| 74 | RALGDS |  |  | 770 | 0.621 | 0.4326 | Yes |
| 75 | TMEM108 |  |  | 772 | 0.619 | 0.4365 | Yes |
| 76 | ZNF711 |  |  | 774 | 0.618 | 0.4404 | Yes |
| 77 | LRRTM2 |  |  | 776 | 0.617 | 0.4443 | Yes |
| 78 | KIDINS220 |  |  | 777 | 0.616 | 0.4483 | Yes |
| 79 | ST3GAL6 |  |  | 785 | 0.610 | 0.4510 | Yes |
| 80 | MAPT |  |  | 844 | 0.583 | 0.4435 | No |
| 81 | STMN4 |  |  | 876 | 0.570 | 0.4412 | No |
| 82 | ZNF195 |  |  | 883 | 0.568 | 0.4438 | No |
| 83 | PLPPR5 |  |  | 941 | 0.546 | 0.4362 | No |
| 84 | MXI1 |  |  | 944 | 0.546 | 0.4394 | No |
| 85 | GDPD1 |  |  | 946 | 0.545 | 0.4428 | No |
| 86 | EIF1B |  |  | 978 | 0.533 | 0.4403 | No |
| 87 | GDI1 |  |  | 1002 | 0.521 | 0.4392 | No |
| 88 | CLASP2 |  |  | 1017 | 0.515 | 0.4399 | No |
| 89 | EVL |  |  | 1047 | 0.506 | 0.4375 | No |
| 90 | NELFCD |  |  | 1053 | 0.504 | 0.4399 | No |
| 91 | OLFM1 |  |  | 1064 | 0.502 | 0.4413 | No |
| 92 | GABRB3 |  |  | 1151 | 0.479 | 0.4276 | No |
| 93 | TSPAN7 |  |  | 1181 | 0.471 | 0.4250 | No |
| 94 | KLC1 |  |  | 1196 | 0.466 | 0.4253 | No |
| 95 | PARP6 |  |  | 1203 | 0.464 | 0.4272 | No |
| 96 | LMO3 |  |  | 1206 | 0.463 | 0.4299 | No |
| 97 | AUTS2 |  |  | 1211 | 0.462 | 0.4322 | No |
| 98 | IRS2 |  |  | 1218 | 0.458 | 0.4340 | No |
| 99 | GNG4 |  |  | 1230 | 0.457 | 0.4349 | No |
| 100 | H1FX |  |  | 1238 | 0.454 | 0.4365 | No |
| 101 | NBEA |  |  | 1239 | 0.454 | 0.4395 | No |
| 102 | ASRGL1 |  |  | 1258 | 0.451 | 0.4390 | No |
| 103 | PHYHIPL |  |  | 1270 | 0.447 | 0.4398 | No |
| 104 | KIF1A |  |  | 1304 | 0.440 | 0.4362 | No |
| 105 | ADAM22 |  |  | 1307 | 0.439 | 0.4387 | No |
| 106 | RTN2 |  |  | 1333 | 0.433 | 0.4367 | No |
| 107 | KIAA1211 |  |  | 1346 | 0.428 | 0.4372 | No |
| 108 | SLC10A4 |  |  | 1356 | 0.427 | 0.4382 | No |
| 109 | BMP7 |  |  | 1368 | 0.423 | 0.4389 | No |
| 110 | TUBB2A |  |  | 1373 | 0.422 | 0.4409 | No |
| 111 | RIMS3 |  |  | 1418 | 0.412 | 0.4350 | No |
| 112 | RNFT2 |  |  | 1426 | 0.410 | 0.4363 | No |
| 113 | CHGB |  |  | 1466 | 0.398 | 0.4313 | No |
| 114 | PEG3 |  |  | 1514 | 0.386 | 0.4247 | No |
| 115 | ARHGEF7 |  |  | 1536 | 0.381 | 0.4231 | No |
| 116 | RIMBP2 |  |  | 1576 | 0.371 | 0.4179 | No |
| 117 | TMEM178B |  |  | 1639 | 0.360 | 0.4081 | No |
| 118 | ZNF24 |  |  | 1671 | 0.355 | 0.4044 | No |
| 119 | FSD1 |  |  | 1701 | 0.349 | 0.4010 | No |
| 120 | PRSS12 |  |  | 1704 | 0.349 | 0.4029 | No |
| 121 | RAB33A |  |  | 1760 | 0.337 | 0.3944 | No |
| 122 | PHF21B |  |  | 1855 | 0.323 | 0.3781 | No |
| 123 | KLF13 |  |  | 1871 | 0.318 | 0.3773 | No |
| 124 | ELAVL2 |  |  | 1938 | 0.307 | 0.3664 | No |
| 125 | ABCA3 |  |  | 1982 | 0.300 | 0.3599 | No |
| 126 | PBX3 |  |  | 1995 | 0.297 | 0.3595 | No |
| 127 | GLDC |  |  | 2077 | 0.286 | 0.3456 | No |
| 128 | FKBP1B |  |  | 2122 | 0.281 | 0.3388 | No |
| 129 | SLC35G2 |  |  | 2171 | 0.272 | 0.3312 | No |
| 130 | C11orf95 |  |  | 2267 | 0.260 | 0.3143 | No |
| 131 | SNAP25 |  |  | 2289 | 0.257 | 0.3119 | No |
| 132 | AKAP1 |  |  | 2384 | -0.256 | 0.2952 | No |
| 133 | UCP2 |  |  | 2602 | -0.283 | 0.2545 | No |
| 134 | PDK1 |  |  | 2754 | -0.301 | 0.2270 | No |
| 135 | TBPL1 |  |  | 3004 | -0.333 | 0.1804 | No |
| 136 | MSH6 |  |  | 3051 | -0.339 | 0.1736 | No |
| 137 | GATA3 |  |  | 3134 | -0.351 | 0.1599 | No |
| 138 | CHML |  |  | 3236 | -0.366 | 0.1425 | No |
| 139 | MCM2 |  |  | 3240 | -0.366 | 0.1443 | No |
| 140 | RNF144A |  |  | 3276 | -0.371 | 0.1399 | No |
| 141 | NET1 |  |  | 3471 | -0.404 | 0.1046 | No |
| 142 | CYGB |  |  | 3579 | -0.423 | 0.0864 | No |
| 143 | CDC42EP3 |  |  | 3608 | -0.430 | 0.0838 | No |
| 144 | FKBP4 |  |  | 3632 | -0.433 | 0.0821 | No |
| 145 | KNSTRN |  |  | 3646 | -0.435 | 0.0825 | No |
| 146 | MRPL48 |  |  | 4027 | -0.519 | 0.0115 | No |
| 147 | SEPT6 |  |  | 4051 | -0.526 | 0.0104 | No |
| 148 | ZWILCH |  |  | 4082 | -0.534 | 0.0081 | No |
| 149 | FOXM1 |  |  | 4158 | -0.556 | -0.0029 | No |
| 150 | MCM6 |  |  | 4273 | -0.590 | -0.0214 | No |
| 151 | NFIL3 |  |  | 4309 | -0.600 | -0.0242 | No |
| 152 | KIF15 |  |  | 4324 | -0.603 | -0.0230 | No |
| 153 | RFC4 |  |  | 4340 | -0.611 | -0.0219 | No |
| 154 | GLRX |  |  | 4374 | -0.626 | -0.0242 | No |
| 155 | RBBP8 |  |  | 4375 | -0.627 | -0.0201 | No |
| 156 | FIGNL1 |  |  | 4411 | -0.639 | -0.0227 | No |
| 157 | CSE1L |  |  | 4419 | -0.642 | -0.0199 | No |
| 158 | DDX39A |  |  | 4467 | -0.661 | -0.0247 | No |
| 159 | POPDC3 |  |  | 4522 | -0.691 | -0.0307 | No |
| 160 | C4orf48 |  |  | 4536 | -0.698 | -0.0286 | No |
| 161 | SYT1 |  |  | 4552 | -0.707 | -0.0269 | No |
| 162 | TUBB4B |  |  | 4575 | -0.722 | -0.0264 | No |
| 163 | PBK |  |  | 4593 | -0.730 | -0.0249 | No |
| 164 | BIRC5 |  |  | 4618 | -0.745 | -0.0247 | No |
| 165 | MIAT |  |  | 4622 | -0.748 | -0.0204 | No |
| 166 | NUSAP1 |  |  | 4654 | -0.765 | -0.0214 | No |
| 167 | GGCT |  |  | 4666 | -0.772 | -0.0184 | No |
| 168 | MCM7 |  |  | 4704 | -0.800 | -0.0204 | No |
| 169 | RANBP1 |  |  | 4741 | -0.823 | -0.0220 | No |
| 170 | VRK1 |  |  | 4773 | -0.842 | -0.0225 | No |
| 171 | NUF2 |  |  | 4782 | -0.847 | -0.0185 | No |
| 172 | PRIM1 |  |  | 4815 | -0.881 | -0.0189 | No |
| 173 | DKK1 |  |  | 4828 | -0.893 | -0.0154 | No |
| 174 | PRC1 |  |  | 4846 | -0.912 | -0.0127 | No |
| 175 | GMNN |  |  | 4873 | -0.939 | -0.0116 | No |
| 176 | NGRN |  |  | 4879 | -0.944 | -0.0063 | No |
| 177 | DNAJC9 |  |  | 4916 | -0.982 | -0.0069 | No |
| 178 | L1CAM |  |  | 4924 | -0.992 | -0.0017 | No |
| 179 | GAL |  |  | 4937 | -1.001 | 0.0026 | No |
| 180 | UBE2T |  |  | 5000 | -1.091 | -0.0023 | No |
| 181 | CDCA5 |  |  | 5023 | -1.133 | 0.0008 | No |
| 182 | MYBL2 |  |  | 5041 | -1.172 | 0.0052 | No |
| 183 | UBE2C |  |  | 5106 | -1.314 | 0.0014 | No |
| 184 | CENPU |  |  | 5159 | -1.486 | 0.0010 | No |
| 185 | HMGA1 |  |  | 5220 | -1.792 | 0.0011 | No |
| 186 | RRM2 |  |  | 5232 | -1.875 | 0.0114 | No |
Table: GSEA details [plain text format]

  

Fig 2: VANGRONINGEN\_ADRN: Random ES distribution      
 Gene set null distribution of ES for **VANGRONINGEN\_ADRN**
